# The grapevine VvibZIPC22 transcription factor is involved in the regulation of flavonoid biosynthesis

**DOI:** 10.1093/jxb/erw181

**Published:** 2016-05-18

**Authors:** Giulia Malacarne, Emanuela Coller, Stefan Czemmel, Urska Vrhovsek, Kristof Engelen, Vadim Goremykin, Jochen Bogs, Claudio Moser

**Affiliations:** ^1^Research and Innovation Centre, Fondazione Edmund Mach, Via E. Mach 1, 38010 S. Michele all’Adige, Trento, Italy; ^2^Centre for Organismal Studies Heidelberg, University of Heidelberg, Im Neuenheimer Feld 360, D-69120 Heidelberg, Germany; ^3^Studiengang Weinbau und Oenologie, Dienstleistungszentrum Laendlicher Raum Rheinpfalz, Breitenweg 71, D-67435 Neustadt, Germany

**Keywords:** bZIP factors, co-expression analysis, flavonoids, gene expression, grapevine, luciferase assay, metabolic profiling, tobacco, transient expression.

## Abstract

In grapevine, flavonoids constitute one of the most abundant subgroups of secondary metabolites, influencing the quality, health value, and typicity of wines. Their synthesis in many plant species is mainly regulated at the transcriptional level by modulation of flavonoid pathway genes either by single regulators or by complexes of different regulators. In particular, bZIP and MYB factors interact synergistically in the recognition of light response units present in the promoter of some genes of the pathway, thus mediating light-dependent flavonoid biosynthesis. We recently identified *VvibZIPC22*, a member of clade C of the grapevine bZIP family, in a quantitative trait locus (QTL) specifically associated with kaemperol content in mature berries. Here, to validate the involvement of this candidate gene in the fine regulation of flavonol biosynthesis, we characterized its function by *in vitro* and *in vivo* experiments. A role for this gene in the control of flavonol biosynthesis was indeed confirmed by its highest expression at flowering and during UV light-mediated induction, paralleled by accumulation of the flavonol synthase 1 transcript and flavonol compounds. The overexpression of *VvibZIPC22* in tobacco caused a significant increase in several flavonoids in the flower, via induction of general and specific genes of the pathway. In agreement with this evidence, VvibZIPC22 was able to activate the promoters of specific genes of the flavonoid pathway, alone or together with other factors, as revealed by transient reporter assays. These findings, supported by *in silico* indications, allowed us to propose VvibZIPC22 as a new regulator of flavonoid biosynthesis in grapevine.

## Highlight

Functional analysis of the grapevine VvibZIPC22 indicates that this factor is involved in the regulation of different branches of the flavonoid pathway, either alone or together with other factors.

## Introduction

In grapevine (*Vitis vinifera* L.), as well as in many plant species, flavonoids represent one of the most abundant subgroups of phenolic compounds, whose synthesis derives from the amino acid phenylalanine through the phenylpropanoid pathway. They are composed of three main classes, anthocyanins, proanthocyanidins (also known as condensed tannins), and flavonols. They accumulate preferentially in flowers and in the peripheral layers of the berry pericarp and of the seed coat of grapes, and fulfil several important functions. They (i) mediate the response to biotic and abiotic stresses (temperature, UV light, nutrition, water deficit); and (ii) provide pigmentation to flowers and fruits, thereby also influencing the quality and typicity of wines. Moreover, they are associated with health-promoting effects ascribed to grape-rich diets including wine (Flamini *et al.*, 2013; Teixeira *et al.*, 2013).

Due to this wide range of functions, the synthesis of these compounds is finely regulated, mainly at the transcriptional level. The expression of the flavonoid biosynthetic genes is often regulated at the organ- and stage-specific level during berry development, thus leading to the accumulation of specific flavonoids in the different organs and phases. Although flavonoid and especially anthocyanin biosynthesis has been extensively studied, also in grapevine, its regulation is not yet completely understood (Hichri *et al.*, 2011a). The whole system is under the tight control of several transcription factors, which can mutually interact giving rise to regulatory specificity (Grotewold, 2006). In all species analysed to date, these factors mainly belong to the family of the R2R3-MYB domain-containing proteins (Hichri *et al.*, 2011a; Li, 2014). However, there is evidence which indicates bZIP proteins as involved together with MYB and other factors in the transcriptional control of flavonoid biosynthesis depending on light (Hartmann *et al.*, 2005; Czemmel *et al.*, 2009; Stracke *et al.*, 2010).

bZIP factors belong to a large family already characterized in many plant species, including grapevine (Liu *et al.*, 2014). These factors are involved in the regulation of plant development (Strathmann *et al.*, 2001), response to drought (Yoshida *et al.*, 2010), high salinity (Seong *et al.*, 2008), light (Hartmann *et al.*, 2005; Stracke *et al.*, 2010), and pathogen infection (Thurow *et al.*, 2005), as well as amino acid (Hanson *et al.*, 2008; Dietrich *et al.*, 2011) and phenylpropanoid biosynthesis (Heinekamp *et al.*, 2002; Hartmann *et al.*, 2005). So far, only a few grapevine *bZIP* genes have been characterized, mainly as being involved in the abscisic acid (ABA)-dependent response to abiotic stresses (Tak and Mhatre, 2013) and grape berry ripening (Nicolas *et al.*, 2014).

Plant bZIPs bind to ACGT-containing elements (Izawa *et al.*, 1993) and usually regulate transcription by forming dimers (Strathmann *et al.*, 2001; Ehlert *et al.*, 2006) and interacting with non-bZIP proteins (Schutze *et al.*, 2008), to gain flexibility in their regulatory capacity. In particular, bZIP heterodimers of the C/S1 groups of Arabidopsis have been implicated in stress response and development (Weltmeier *et al.*, 2006) and in sugar-regulated control of amino acid metabolism during the plant response to starvation (Dietrich *et al.*, 2011).

Flavonoid biosynthesis in grapevine is also regulated by environmental stimuli (Teixeira *et al.*, 2013) and is particularly affected by light (Fujita *et al.*, 2006; Matus *et al.*, 2009; Koyama *et al.*, 2012). Koyama *et al.* (2012) described the influence of light quality on the regulation of flavonoids in young berry skins: visible light primarily induces proanthocyanidin (PA) biosynthesis, whereas UV light speciﬁcally induces ﬂavonol biosynthesis. In Arabidopsis, the light regulation of flavonol biosynthesis is directed by MYB and bZIP factors which co-operatively bind to the light regulatory units (LRUs) present in the *CHS* and *FLS* promoter sequence (Hartmann *et al.*, 2005). A well-studied example is the Arabidopsis *HY5* gene, encoding a bZIP factor which regulates numerous genes, such as *AtCHS*, *AtFLS*, and *AtMYB12*, during photomorphogenesis (Lee *et al.*, 2007; Stracke *et al.*, 2010). The presence of LRUs in the promoters of *VviFLS1* and *VviMYBF1* and their light responsiveness led Czemmel *et al.* (2009) to propose them as targets of a grapevine HY5 homologue.

Recently, we have identified two grapevine *bZIP* genes, namely the predicted *VvbZIP14* and *VvbZIP22* (Liu *et al.*, 2014), within two quantitative trait locus (QTL) regions associated with the fine tuning of flavonol content in mature grapes, on chromosomes 5 and 7, respectively. Notably, these bZIPs were also expressed at different levels in grapes of ‘Syrah’×‘Pinot Noir’ progeny showing different contents of flavonols in the mature skin (Malacarne *et al.*, 2015). The QTL on chromosome 7, containing *VvbZIP22*, was specifically related to kaempferol content, while the other, containing *VvbZIP14*, was also associated with anthocyanin content (Costantini *et al.*, 2015). Starting from this evidence, in this study, we decided to characterize further *VvbZIP22*, here renamed *VvibZIPC22* according to the recently proposed grapevine genome nomenclature system (Grimplet *et al.*, 2014).

## Materials and methods

### Grapevine material and sampling

Inflorescences and clusters from a representative sample of *V. vinifera* cv. Pinot noir (clone ENTAV115) were collected at six developmental stages during the 2011 season at the Giaroni experimental field of FEM (Edmund Mach Foundation, San Michele a/Adige, Italy, 46°18'N, 11°13'E). To be more representative, different parts of each inflorescence/cluster were collected from shady and sunny sides. The six stages corresponded to flowering (50% opened flowers, E-L 23), pepper corn (E-L 29), pre-véraison (hard green berries, E-L 33), véraison (50% coloured berries, E-L 35), post-véraison (berries at 16° Brix, E-L 36), and maturity (berries at 18° Brix, E-L 38). During sampling, skins were separated from flesh and seeds directly in the field when feasible, precisely from véraison to maturity stages, and immediately frozen in liquid nitrogen.

In the 2012 season, vegetative organs (young leaf, mature leaf, bud, root, internode, and tendril), germinated seeds, green (E-L 20) and mature inflorescences (E-L 23) (divided into calyptra, stamen, and pistillum), and berries at different stages (E-L 29, 34, and 36) were sampled from the same Pinot Noir plants considered in 2011 and immediately frozen in liquid nitrogen. In the case of berries at stages E-L 34 and 36, seeds were removed during grinding. For each stage and organ of the panel, three different plants (biological replicates) were considered.

For light induction experiments, dormant hardwood cuttings of grapevine *V. vinifera* L. cv. Chardonnay were collected from a vineyard in Neustadt/W, Germany (49°22'9''S, 8°10'28''E). Cuttings were grown in sterile Perlite (Knauf, Sittingbourne, UK) under the following conditions: temperature, 25 °C; white light, ~100 µmol m^−2^ s^−1^; 9h light cycle. In this hydroponic system, the apical buds burst after 10 d and roots developed after 4 weeks. After 19 d, the rooted plants were subjected to light treatment [4% UV-B and 30% UV-A light (18W, 6500K)] under the following conditions: temperature, 23 °C; 9h light cycle. In the case of treated samples, the third and the fourth leaf from the shoot tip of five plants were collected at 0, 10, 24, 48, and 72h after the onset of light exposure. Control samples were collected at the same time points, from five other plants grown under the same conditions, but without UV light. At each time point, leaves were pooled, immediately frozen in liquid nitrogen, and stored at –80 °C till use.

All the collected samples were finely ground by liquid nitrogen using frozen metal grinding jars mounted on a Mixer Mill MM 400 (Retsch, Haan, Germany). The powder was maintained at –80 °C maximum for 2 months to be used for both transcriptional and biochemical analyses.

### Cloning of *VvibZIPC22* and tobacco transformation

The cDNA from Pinot Noir berry skins at the véraison stage diluted 10-fold was used for PCR amplification with forward primer (bZIP-XhoI 5'-CTCGAGGAAAATGTCAGCGATGCAAC-3', *Xho*I recognition site underlined) and reverse primer (bZIP-KpnI 5'-GGTACCTCAGCACTGAAACATGTTTG-3', *Kpn*I recognition site underlined). The *VvibZIPC22* sequence considered in this study was derived from the 12Xv1 assembly of the Pinot Noir genome (VIT_07s0005g01450, from http://genomes.cribi.unipd.it/DATA/V1, last accessed 11 December 2015) and confirmed by EST sequences (EE065899.1, EC946004.1, and EC945791.1). The complete coding DNA sequence (CDS) of *VvibZIPC22* was amplified in a 12.5 µl PCR mix containing primers (200 pM each), 1U of Phusion DNA polymerase (Thermo Fisher Scientific, Waltham, MA, USA), dNTPs (200 µM), PCR buffer (1×), and cDNA (diluted 10-fold). The PCR conditions adopted were: 95 °C for 2min, followed by 35 cycles, each one consisting of 95 °C for 30s, 58 °C for 30s, and 72 °C for 45s, followed by a final extension (7min at 72 °C). A PCR product of 438bp was obtained and subcloned into a pGEM-T Easy Vector (Promega, Mannheim, Germany) for sequencing. The *VvibZIPC22* CDS (KX073969) was subsequently transferred into an *Xho*I/*Kpn*I-digested pART7 primary cloning vector (Gleave, 1992) under the control of the *Cauliflower mosaic virus* (CaMV) 35S promoter to give pART7bZIPC22. In both cases, a chemical transformation was performed using One Shot^®^ TOP10 competent *Escherichia coli* cells (Thermo Fisher Scientific).

pART7bZIPC22 was used both for the transient expression assays and as an intermediate to obtain pART27bZIPC22 for tobacco transformation. *CaMV35S:VvibZIPC22* was therefore transferred into a *Not*I-digested pART27 binary vector to obtain pART27bZIPC22. Single colonies of *Agrobacterium tumefaciens* (strain C58C1) transformed with pART27bZIPC22 by electroporation were cultured in 50ml of selective YEB medium and grown overnight (28 °C at 160rpm). The cells were harvested by centrifugation at 4000rpm for 30min without break and resuspended in 50ml of infiltration buffer (10mM MES, pH 5.9 and 150 μM acetosyringone). The suspension was incubated at 28 °C and 50rpm for 2h. Thereafter the concentration was measured by spectroscopy (OD_600 nm_) and adjusted to a final concentration of OD_600 nm_ of 0.3 with H_2_O. *Nicotiana tabacum* cv. Samsung leaf sections (1cm^2^) were further incubated with the bacterial suspension right-side up for 30min at room temperature. Leaf explants were transferred to a sterile wet filter paper and incubated in the dark for 2 d at room temperature. The explants were then transferred right-side down for 30min to solid Murashige and Skoog (MS) medium plus timentin (400mg l^–1^), supplemented with B5 vitamins, 3% (w/v) sucrose, 1mg l^–1^ 6-benzyladenine (BA), 0.1mg l^–1^ α-naphthaleneacetic acid (NAA), 0.8% tissue culture agar, pH 5.6, and 100mg l^–1^ kanamycin. Explants were then incubated in the dark for 2 weeks at 25 °C, and then transferred to 25 °C, 16h light cycle. Finally, kanamycin-resistant plantlets were transferred to soil mix, acclimatized, and grown in the greenhouse. The presence of the transgene in transformed lines was checked by PCR with the primers 35SCaMVpART7 (5'-CAATCCCACTATCCTTCGCA-3') and bZIP-KpnI. Stamens and petal limbs at two developmental stages (open flower without pollen and mature flower) were collected from a representative sample of each transgenic line and wild-type plant and stored at –80 °C until biochemical and gene expression analyses.

### Grapevine transient expression experiments

A transient expression system using Chardonnay liquid cell cultures was established as described previously (Bogs *et al.*, 2007). Cells were bombarded with 1.6 µm gold particles using a PDS-1000/He Biolistic Particle Delivery System from Bio-Rad (München, Germany) with 4481 kPa helium pressure, a vacuum of 86 kPa, and a distance of 9.5cm. The luciferase assay was performed as set up by Czemmel *et al.* (2009). Briefly, gold particles were coated with 500ng of each respective plasmid, giving a total amount of 2 µg of plasmid per transformation for reporter/effector bombardments. Additionally, each bombardment contained a positive control of 100ng of the *Renilla* luciferase plasmid pRluc (Horstmann *et al.*, 2004). After incubation in the dark at 27 °C for 48h, the harvested cells were assayed for luciferase activities using the dual-luciferase reporter assay system (Promega) measured with a Lumat LB 9507 Luminometer (Berthold Technologies, Bad Wildbad, Germany). The relative luciferase activity was calculated as the ratio between the ﬁreﬂy and the *Renilla* (control) luciferase activities after subtraction of the cell background (ground cells but not bombarded). All transfection experiments were carried out in triplicate, and each promoter experiment was repeated at least three times.

The design of the effector constructs of VviMYBPA1 (AM259485) and VviMYBA2 (BAD18978), VviMYBF1 (ACV81697), and VviMYC1 (ACC68685) is described in Bogs *et al.* (2007), Czemmel *et al.* (2009), and Hichri *et al.* (2010), respectively. The effector construct of VvibZIPC22 (pART7bZIPC22) was obtained as previously described.

The cloning of the promoter fragments of *VviCHI* (X75963), *VviANR* (CAD91911), and *VviUFGT* (AY955269), *VviFLS1* (FJ948478), into the luciferase reporter vector pLuc (Horstmann *et al.*, 2004) is described in Bogs *et al.* (2007) and Czemmel *et al.* (2009), respectively. The same approach was adopted to clone a 1100bp fragment of *VviCHS1* (AB015872), *VviCHS2* (AB066275), and *VviCHS3* (AB066274) promoters by specific primers, whose sequences were as follows, with restriction sites for *Sac*I and *Xho*I underlined: CHS1pF (5'-TATGAGCTCATCTAACACCGCTGGGACT-3'), CHS1pR (5'-TATCTCGAGTGTTGGCTACCTGCTTCAC-3'), CHS2pF (5'-TATGAGCTCTTGGTGAGAGATTAAAGGGAAA-3'), CHS2pR (5'-TATCTCGAGTTTTGGCTGCTTGAATCAGTGT-3'), CHS3pF (5'-TATGAGCTCGTGCCATAATTGCCTCAAA-3'), and CHS3pR (5'-TATCTCGAGCTTCAGAGGGATGAGGCTTG-3').

### Biochemical analyses of flavonoids in grapevine and tobacco samples

Extraction of anthocyanins, flavonol aglycons, and flavan-3-ol monomers and their analysis by HPLC-DAD (diode array detection) from grapevine samples were performed as described in Mattivi *et al.* (2006, 2009) using a Waters 2690 HPLC system equipped with Waters 996 DAD and Empower software (Waters Corporation, Milford, MA, USA). Some modifications were applied to the extraction of flavonols from light-treated and control leaf samples. Briefly, three separate 200mg aliquots (technical replicates) of frozen powder were extracted with 1.5ml of methanol by rotation (30rpm for 20min). After centrifugation (13 000rpm for 5min), 1.5ml of the supernatant was transferred to a 50ml flask. The same procedure was adopted in a second extraction with 1.5ml of methanol. The flask with a total volume of 10ml (3ml of methanolic extract+2ml of methanol+5ml of trifluoroacetic acid 2M in water) was used for the following acid hydrolysis as in Mattivi *et al.* (2006).

Extraction of anthocyanins and flavonols from tobacco stamens was performed starting from three separate 150mg aliquots (technical replicates) of frozen powder. After the addition of 1.5ml of methanol, each sample was shaken at room temperature for 20min. After centrifugation (14 000rpm for 5min), each sample was then filtered with 0.22 µm filters into LC vials and subjected to HPLC-DAD analysis.

Each anthocyanin, flavonol, and flavan-3-ol compound was identified by comparison of its retention time and UV spectra at 520, 370, and 280nm. The concentration (µg g FW^−1^) of each compound was determined using the external standard method, specific for each compound. Values under the limit of detection (LOD) and of quantification (LOQ) were given a value equal to zero in the final quantification.

Proanthocyanidins (PAs) in tobacco petals were determined using dimethylaminocinnamaldehyde (DMACA) reagent described by Nagel and Glories (1991). Briefly, three separate 150mg aliquots (technical replicates) of frozen powder were extracted with 2ml of AA-buffer (70% acetone, 0.1% ascorbate) by sonication in ice-water (20kHz, amplitude 50% for 30s) with a Sonifier SFX250 (Branson Ultrasonics, Danbury, CT, USA). After centrifugation (13 000rpm, 4 °C), 1ml of extract was mixed with 0.75ml of diethylether to remove chlorophyll, and stored at –20 °C for 2h. After incubation, 100 µl of the lower PA phase was mixed with 900 µl of a DMACA solution (0.1% DMACA, 1% 3N HCl) and incubated in the dark (room temperature for 60min). Finally, the PA concentration (µg g FW^−1^) was determined reading OD_640 nm_ against a blank DMACA solution and using catechin (Sigma Aldrich, St. Louis, MO, USA) as standard.

### Quantitative gene expression analysis in grapevine and tobacco samples

Total RNA was isolated from grapevine and tobacco samples with the Spectrum Plant Total RNA Kit (Sigma Aldrich) according to the manufacturer’s instructions. RNA was quantified with an ND-8000 nanodrop spectrophotometer and checked for integrity with a 2001-Bioanalyzer (Agilent Technologies, Santa Clara, CA, USA). For reverse transcription–PCR and real-time RT–PCR (qRT-PCR) analyses, cDNAs were synthesized using the SuperscriptVILO™ cDNA Synthesis Kit from 1.5 μg of DNAse I-treated RNA (Thermo Fisher Scientific), following the manufacturer’s instructions. qRT-PCR analyses were carried out using the Platinum SYBR Green qPCR SuperMix-UDG in a ViiA™ 7 thermocycler (Thermo Fisher Scientific). The 384-well plates were set up according to the sample maximization strategy proposed in Hellemans *et al.* (2007), and each reaction was run in triplicate. Reaction conditions and the protocol of analysis were the same as adopted in Malacarne *et al.* (2015). Grapevine and tobacco housekeeping genes were tested for their stability in each experiment using the GeNorm software (Vandesompele *et al.*, 2002). Normalized relative quantities (NRQs) were then calculated by dividing RQs by a normalization factor, based on the expression of the most stable reference genes (details are given in the figure legends) (Reid *et al.*, 2006). Primer sequences used for the analysis are listed in Supplementary Table S1 at *JXB* online, as designed in this work or taken from the literature.

### Bioinformatics

For the research of plant *cis*-regulatory elements, the first 1000bp from the putative transcriptional start of the promoter sequences were screened manually and by the use of PLACE (http://www.dna.affrc.go.jp/PLACE/; Higo *et al.*, 1999).

For phylogenetic studies, the complete CDS of *VvibZIP* genes belonging to clade B and C (Liu *et al.*, 2014) and of their putative orthologues in different plant species were retrieved from public databases (http://genomes.cribi.unipd.it/DATA/V1 and http://www.ncbi.nlm.nih.gov/, last accessed 11 December 2015). Codon-based alignments of *bZIP* CDS sequences were made using the Macse program (Ranwez *et al.*, 2011). Automatically produced alignments were loaded into the Seaview alignment editor and manually edited in amino acid mode to discard misaligned regions. The final selection of alignment columns was saved to produce (i) a 1335 positions long curated alignment of coding clade B *VvibZIP* sequences and their putative orthologues (Supplementary Dataset S1) and (ii) a 297 positions long curated alignment of coding clade C *VvibZIP* sequences and their putative orthologues (Supplementary Dataset S2). Bayesian analyses were performed employing the MPI version of Phylobayes (version 1.4) and a general specification of the CAT+GTR+G4 model. Two chains were run on each data set under each model and until 5000 cycles were sampled. Trees were built for each data set based on the two chains, discarding the first 2500 cycles as burn in, which was sufficient for ML (maximum likelihood) parameter values to maximize in all analyses. Parameter values were then sampled every cycle thereafter till the 5000th cycle.

Co-expression analyses were performed by means of the Vitis Expression Studies Platform Using COLOMBOS Compendia Instances (VESPUCCI) (M. Moretto *et al*., 2016*b*), a grapevine gene expression compendium (http://vespucci.colombos.fmach.it/) based on COLOMBOS v3.0 technology (Moretto *et al*., 2016*a*). The analyses by COLOMBOS were essentially based on contrast relevance and gene similarity scores as thoroughly described in Text S1 from Engelen *et al.* (2011) and the updates made with the v3.0 release.

## Results

### *VvibZIPC22* belongs to one group of clade C in the phylogenetic tree of *VvibZIP* factors

The recent phylogenetic analysis of the grapevine *bZIP* gene family assigned *VvbZIP22* to clade C, together with another nine *VvbZIP* genes. In particular, *VvbZIP22*, together with *VvbZIP14* and *VvbZIP37*, forms a homologous triplet with collinearity along chromosomes 5, 7, and 14 (Liu *et al.*, 2014). According to the recently suggested grapevine genome nomenclature system (Grimplet *et al.*, 2014), we renamed the VvbZIP factors indicating the clade to which they belong, with the exception of the unresolved members.

**Fig. 1.**
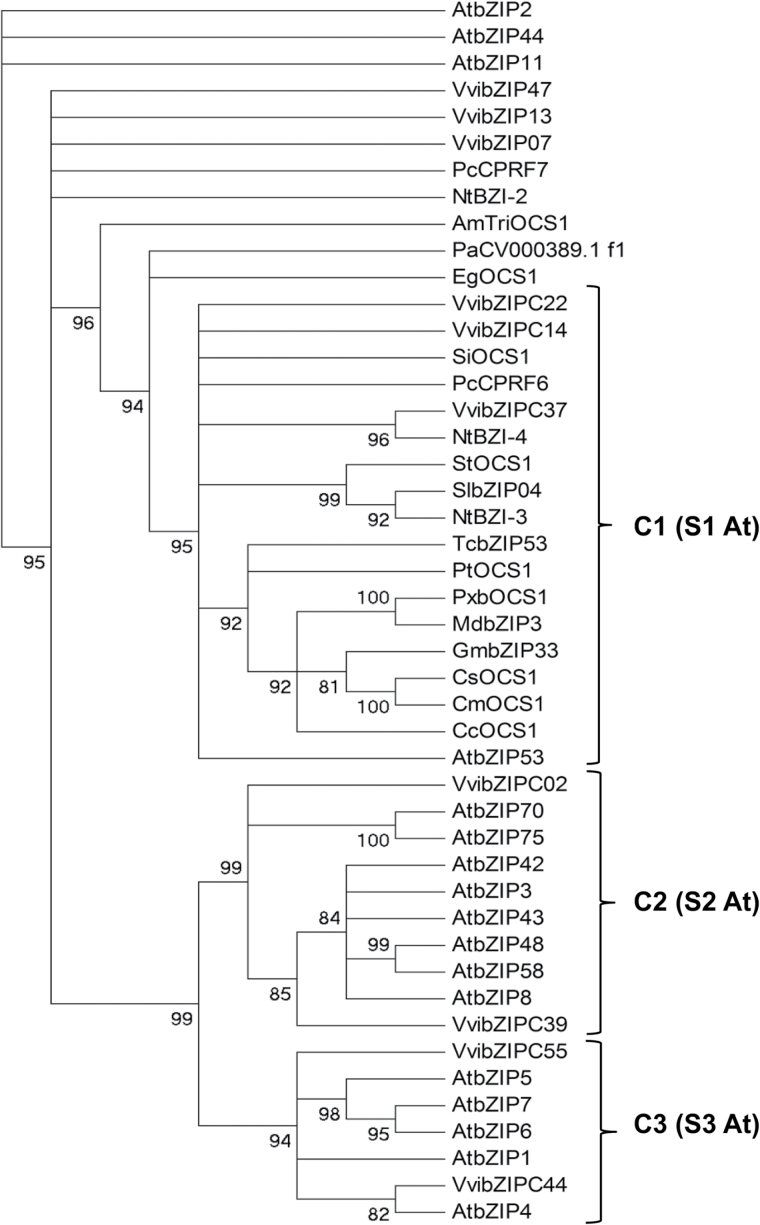
Phylogenetic tree of clade C VvibZIP factors and of their putative orthologues. C1, C2, and C3 correspond to the identified groups within clade C, similarly to those identified in clade S in Arabidopsis (Ehlert *et al.*, 2006). Bootstrap values are shown next to the nodes. Gene names with their accession numbers are the following: *AmTriOCS1* (XM_011623592.1), *AtbZIP1* (NM_124322), *AtbZIP2* (NM_127373), *AtbZIP3* (NM_121588), *AtbZIP4* (NM_104646), *AtbZIP5* (NM_114836), *AtbZIP6* (NM_127850), *AtbZIP7* (NM_119935), *AtbZIP8* (NM_105562), *AtbZIP11* (NM_119625), *AtbZIP42* (NM_113954), *AtbZIP43* (NM_123241), *AtbZIP44* (NM_106193), *AtbZIP48* (NM_126441), *AtbZIP53* (NM_116107), *AtbZIP58* (NM_101230), *AtbZIP70* (NM_125476), *AtbZIP75* (NM_180460), *CcOCS1* (XM_006444430), *CmOCS1* (XM_008456072), *CsOCS1* (XM_004152178), *EgOCS1* (XM_010916264), *GmbZIP33* (XM_003521545), *MdbZIP3* (NM_001294367), *NtBZI-2* (AY045570), *NtBZI-3* (AY045571), *NtBZI-4* (AY045572), *PaCV000389.1_f1* (CV000389), *PcCPRF6* (AJ292744), *PcCPRF7* (AJ292745), *PtOCS1* (XM_002301475), *PxbOCS1* (XM_009341500), *SiOCS1* (XM_011081252), *SlbZIP04* (Solyc01g079480), *StOCS1* (XM_006355774), *TcbZIP53* (XM_007051040), *VvibZIPC02* (VIT_01s0010g00930), *VvibZIP07* (VIT_03s0038g04450), *VvibZIP13* (VIT_04s0023g02430), *VvibZIPC14* (VIT_05s0077g01140), *VvibZIPC22* (KX073969), *VvibZIPC37* (VIT_14s0060g01210), *VvibZIPC39* (VIT_14s0083g00700), *VvibZIPC44* (VIT_18s0001g08710), *VvibZIP47* (VIT_18s0001g13040), and *VvibZIPC55* (VIT_00s0541g00020).

To better define the structure of clade C and to identify sequences closely related to *VvbZIP22* from other species, the CDS sequences of VvbZIP factors belonging to this clade were aligned and compared with those of bZIP factors from other important plants and crops with significant identity (>50%) to VvbZIP22 (Supplementary Dataset S2). Phylogenetic analysis revealed that clade C can be divided into three main groups, designated C1, C2, and C3 (Fig. 1), similarly to clade S in Arabidopsis (Ehlert *et al.*, 2006). In particular, *VvibZIPC22* is part of group C1 together with *VvibZIPC14* and *VvibZIPC37* and with previously characterized *bZIP* genes from flowering species such as *N. tabacum* (*NtBZI-3* and *NtBZI-4*; Strathmann *et al.*, 2001), *Petroselinum crispum* (*PcCPRF6*; Rugner *et al.*, 2001), *Arabidopsis thaliana* (*AtbZIP53*; Dietrich *et al.*, 2011), and *Solanum lycopersicum* (*SlbZIP04*; named *LebZIP2* in Seong *et al.* 2008). Moreover, other orthologues can be considered: *SiOCS1* from *Sesamum indicum*, *TcbZIP53* from *Theobroma cacao*, *PtOCS1* from *Populus trichocarpa*, *PxbOCS1* from *Pyrus*×*bretschneideri*, *MdbZIP3* from *Malus domestica*, *GmbZIP33* from *Glycine max*, *CsOCS1* and *CmOCS1* from *Cucumis sativus* and *Cucumis melo*, and *CcOCS1* from *Citrus clementina*. In our analysis, *VvibZIP07*, *VvibZIP13*, and *VvibZIP47*, which in the tree of Liu *et al.* (2014) belong to clade C, were unresolved with respect to group C1 together with *PcCPRF7* from *P. crispum* and *NtBZI-2* from *N. tabacum*. *VvibZIPC02* and *VvibZIPC39* fall into group C2 together with members of group S2 in *A. thaliana* (*AtbZIP3*, *AtbZIP8*, *AtbZIP42*, *AtbZIP43*, *AtbZIP48*, *AtbZIP58*, *AtbZIP70*, and *AtbZIP75*), while *VvibZIPC44* and *VvibZIPC55* form group C3 together with members of group S3 in *A. thaliana* (*AtbZIP4*, *AtbZIP5*, *AtbZIP6*, and *AtbZIP7*) (Fig. 1).

### *VvibZIPC22* expression is flower specific and correlates with kaempferol and quercetin content during berry development

**Fig. 2.**
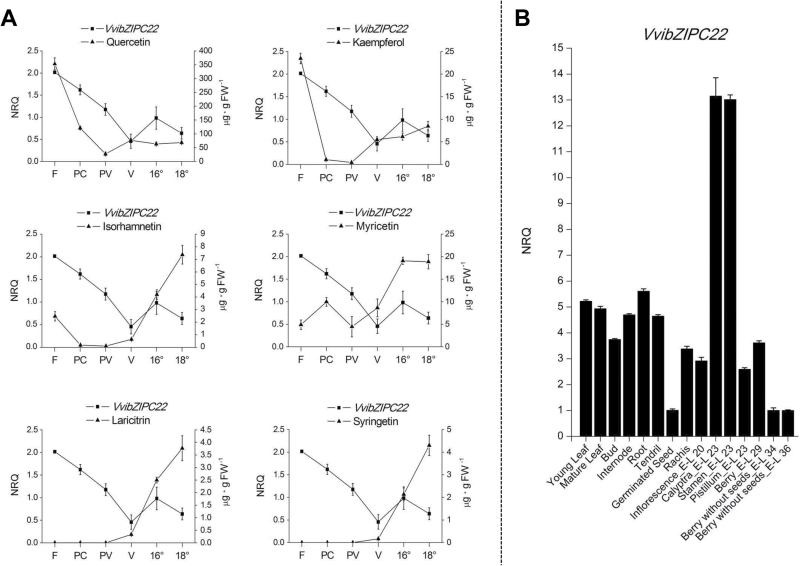
(A) Profiles of *VvibZIPC22* relative expression and of six flavonol aglycons at different Pinot Noir berry developmental stages. (B) *VvibZIPC22* relative expression in a panel of 15 Pinot Noir organs. Transcript levels (NRQs) were determined by qRT-PCR using gene-specific primers (Supplementary Table S1), calibration against the mean expression value in all the stages, and normalization against *VviGADPH* and *VviACTIN* relative expression. Flavonol content was determined by HPLC-DAD analysis. Each value corresponds to the mean and SE of three different biological replicates. F, flowering (50% opened flowers, E-L 23); P, pepper corn (E-L 29); PV, pre-véraison (hard green berries, E-L 33); V, véraison (50% coloured berries, E-L 35); 16°, post-véraison (berries at 16° Brix, E-L 36); 18°, maturity (berries at 18° Brix, E-L 38); NRQ, normalized relative quantity; FW, fresh weight.

To determine if a significant correlation exists between *VvibZIPC22* expression and the content of different flavonoids, we looked at their profiles at six time points of Pinot Noir berry development (Fig. 2A). *VvibZIPC22* expression as well as kaempferol and quercetin aglycons showed similar profiles: they peaked at flowering, reached a minimum between pre-véraison and véraison, and increased slightly towards maturity. It is noteworthy that *VvibZIPC22* expression was highest at flowering, when a peak of expression was also reported for *VviMYBF1* (Czemmel *et al.*, 2009) and *VviFLS1* (Downey *et al.*, 2003) in developing Shiraz berries. The same analysis highlighted that delphinidin-like flavonols had an opposite trend, with maximum accumulation in mature berries (Fig. 2A). Early and late peaks in the biosynthesis of different flavonols have already been described in the *V. vinifera* cv. Pinot Noir (Sternad Lemut *et al.*, 2013). On the other hand, we did not find a significant correlation between *VvibZIPC22* expression and the synthesis of either total anthocyanin 3-monoglucosides or total flavan-3-ol monomers (Supplementary Fig. S1).

The expression analysis extended to different grape organs showed that *VvibZIPC22* is mostly expressed in the flower, as demonstrated by its level in calyptras and stamens, 13 times higher than in berries at stage E-L 36 (16° Brix) and almost three times the expression observed in the other organs (Fig. 2B).

### Induction of *VvibZIPC22* expression by light is related to flavonol accumulation

**Fig. 3.**
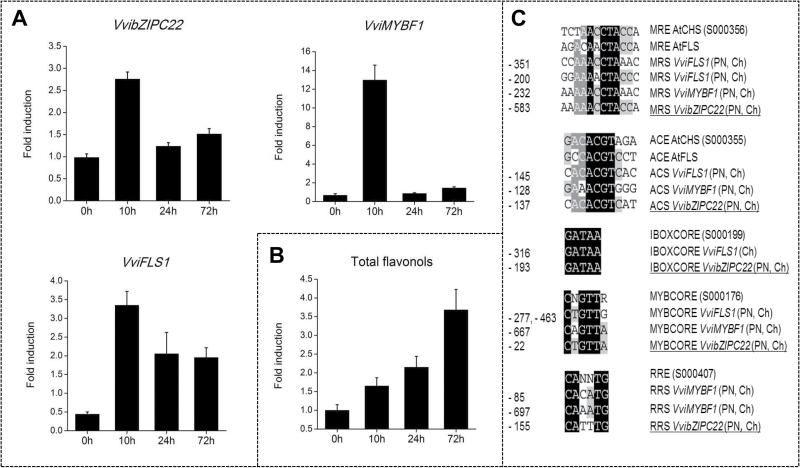
UV light responsiveness of *VvibZIPC22* and flavonol pathway genes. (A) Induction of *VvibZIPC22*, *VviFLS1*, and *VviMYBF1* in Chardonnay leaves after UV light treatment. Each bar corresponds to the fold induction in gene expression between treated and control leaves as determined by qRT-PCR. Transcript levels were measured using gene-specific primers (Supplementary Table S1), calibration against the expression value in the control sample at 0h post-treatment, and normalization against *VviGADPH* and *VviUbiquitin* relative expression. (B) Accumulation of flavonols in Chardonnay leaves after UV light treatment. Each bar corresponds to the fold induction in the total content of flavonol aglycons between treated and control leaves as detected by HPLC-DAD analysis. For both measurements, each data point corresponds to the mean and SE of three independent extractions of the same biological material. (C) Putative light regulatory elements identified in the promoter of *VvibZIPC22* (PN, Ch) in this study (underlined) and in the promoters of *VviMYBF1* and *VviFLS1* (Czemmel *et al.*, 2009). These *cis*-elements were aligned with the corresponding Arabidopsis elements taken from the PLACE database (Higo *et al.*, 1999) using GeneDoc software (v. 1.6). Nucleotides are labelled from black to light grey according to their conservation. Numbers in front of the alignment indicate the relative distance of each element from the putative transcriptional start site (+1). CHS, chalcone synthase; FLS, flavonol synthase; PN, Pinot Noir; Ch, Chardonnay; ACS, ACGT-containing sequence similar to ACE in the Arabidopsis *CHS* promoter (accession no. S000355); MRS, MYB recognition sequence similar to MRE in the Arabidopsis *CHS* promoter (S000356); MYBCORE (S000176), IBOXCORE element (S000199); RRS, R response sequence similar to RRE in the Arabidopsis *CHS* promoter (S000407).

Light-induced flavonol biosynthesis (Downey *et al.*, 2004) is the result of an increase in *VviMYBF1* and *VviFLS1* expression mediated by light regulatory elements present in both promoters and recognized by MYB and bZIP factors (Czemmel *et al.*, 2009). For this reason, we monitored *VvibZIPC22* as well as *VviMYBF1* and *VviFLS1* gene expression over a 3 d time period together with flavonol accumulation in Chardonnay plants exposed to UV light. Within 10h of light exposure, the transcript level of *VvibZIPC22*, *VviFLS1*, and *VviMYBF1* increased ~2.5-, 7.5-, and 22-fold in treated compared with control leaves. *VvibZIPC22* and *VviFLS1* decreased by almost half at 24h and 72h, and *VviMYBF1* was barely detectable at these times (Fig. 3A). The peak of induction of the three genes at 10h of treatment was followed by the accumulation of flavonols starting from 24h and reaching a maximum at 72h of light induction (Fig. 3B).

The presence of light response *cis*-acting elements in the *VvibZIPC22* promoter sequence in both Pinot Noir and Chardonnay was a further indication of its light responsiveness. Hartmann *et al.* (2005) showed that LRUs formed by MYB recognition elements (MREs) and ACGT-containing elements (ACEs) mediate light responsiveness of the *AtCHS* promoter. Similar elements, designated as ACS (ACGT-containing sequence similar to ACE) and MRS (MYB recognition sequence similar to MRE), were also identified in the promoter region of *VviMYBF1* and *VviFLS1* (Czemmel *et al.*, 2009). In this study, we investigated 1000bp of the sequence of Pinot Noir and Chardonnay *bZIPC22* upstream of the putative transcriptional start site: they were identical, with the exception of a polymorphism at position –303. This was not unexpected since Pinot Noir is the male parent of Chardonnay. Moreover, we found that both promoter sequences contain an ACS element at a position (–137bp) fairly well conserved with the ACE site in *AtCHS* and *AtFLS*, and with the ACS site in *VviFLS1* and *VviMYBF1*. In addition, an MRS site (–583bp), possibly recognized by MYB factors, and an IBOXCORE element (–193bp), involved in binding of MYB factors in light-regulated genes in tomato (Rose *et al.*, 1999), were localized upstream of the ACS site (Fig. 3B), as found in the *VviFLS1* promoter (Czemmel *et al.*, 2009).

### VvibZIPC22 activates promoters of general and specific flavonoid pathway genes

**Fig. 4.**
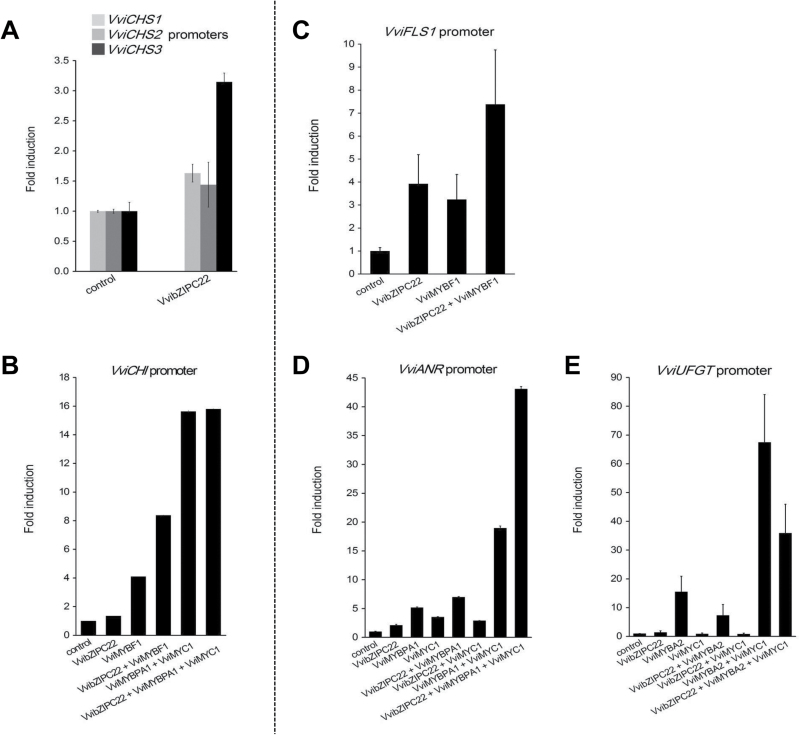
Results of transient reporter assays after bombardment of Chardonnay liquid cell cultures with VvibZIPC22 alone and in combination with characterized grapevine MYB and bHLH factors. Plots on the left of the dashed line (A and B) correspond to the regulators and the promoters of the general flavonoid pathway genes, and plots on the right (C and E) to those of the specific flavonoid pathway genes. Each column represents the fold induction of each specific promoter, plus one or more factors, relative to the respective control (with empty pART7). Mean values and error bars indicating the SE of at least three independent experiments are shown. Each transfection contained the *Renilla* luciferase plasmid pRluc for normalization (Horstmann *et al.*, 2004). CHS, chalcone synthase; CHI, chalcone isomerase; FLS, flavonol synthase; ANR, anthocyanidin reductase; UFGT, UDP-Glc:flavonoid-3-*O*-glucosyltransferase.

In order to identify which ﬂavonoid pathway genes could be the potential targets of VvibZIPC22, transient expression experiments were carried out by bombardment of Chardonnay liquid cell cultures and dual-luciferase assays. Five different promoters were tested: the *VviCHS* and *VviCHI* promoters, as genes controlling the general flavonoid pathway, and the *VviFLS1*, *VviUFGT*, and *VviANR* promoters, as genes specifically involved in the synthesis of flavonols, anthocyanins, and PAs, respectively. VvibZIPC22 was tested alone and in combination with VviMYBF1, VviMYC1–VviMYBPA1, and VviMYBA2 or VviMYC1–VviMYBA2 factors to test the transactivation potential on the *VviCHI* and *VviFLS1* promoters in the first case, on the *VviCHI* and *VviANR* promoters in the second case, and on the *VviUFGT* promoter in the third case (Fig. 4).

VvibZIPC22 was able to activate the promoter of *VviCHS3*, the isoform expressed in red-coloured berry skin during ripening (Goto-Yamamoto *et al.*, 2002), approximately three times more than in the control without VvibZIPC22, suggesting a possible role in the general regulation of the flavonoid pathway (Fig. 4A). VvibZIPC22 also induced the promoter of *VviFLS1* to an extent similar to the positive control VviMYBF1 (~3.5-fold) (Fig. 4C). Interestingly, VvibZIPC22 increased the transactivation potential of VviMYBF1 2-fold by acting synergistically on the promoters of both *VviCHI* (Fig. 4B) and *VviFLS1* (Fig. 4C). As published before (Hichri *et al.*, 2010), VviMYBPA1 in combination with the basic helix–loop–helix (bHLH) factor VviMYC1 induced the *VviANR* promoter (~20-fold), but we observed that its effect is amplified 2-fold by the action of VvibZIPC22, suggesting this bZIP might also play a role in the regulation of PA biosynthesis (Fig. 4D). Conversely, VvibZIPC22 did not display any significant positive effect on the *VviUFGT* promoter either alone or in combination with the complex VviMYC1–VviMYBA2 and with VviMYBA2 (Fig. 4E).

We also noted that the promoters of *VviCHS3*, *VviCHI*, *VviFLS1*, and *VviANR* have an ACGT-containing element in a rather conserved position, between 100bp and 150bp upstream of the transcriptional start site. In contrast, ACGT-containing elements are present only in a more distal position in the promoter of *VviCHS1* and completely absent in those of *VviCHS2* and *VviUFGT* (Supplementary Table S2).

### *VvibZIPC22* overexpression in tobacco leads to flavonoid biosynthesis in different flower organs

The role of VvibZIPC22 *in vivo* was investigated by constitutive overexpression in tobacco plants. Several transgenic lines were generated, propagated, and grown in the greenhouse together with wild-type (WT) plants used for comparison.

We then carried out the following analyses on different flower organs of three WT plants and of six different lines sampled at three development stages: (i) anthocyanin and flavonol content by HPLC-DAD; (ii) PA content by reaction with DMACA; and (iii) gene expression of *VvibZIPC22* and some tobacco flavonoid pathway genes by qRT-PCR.

**Fig. 5.**
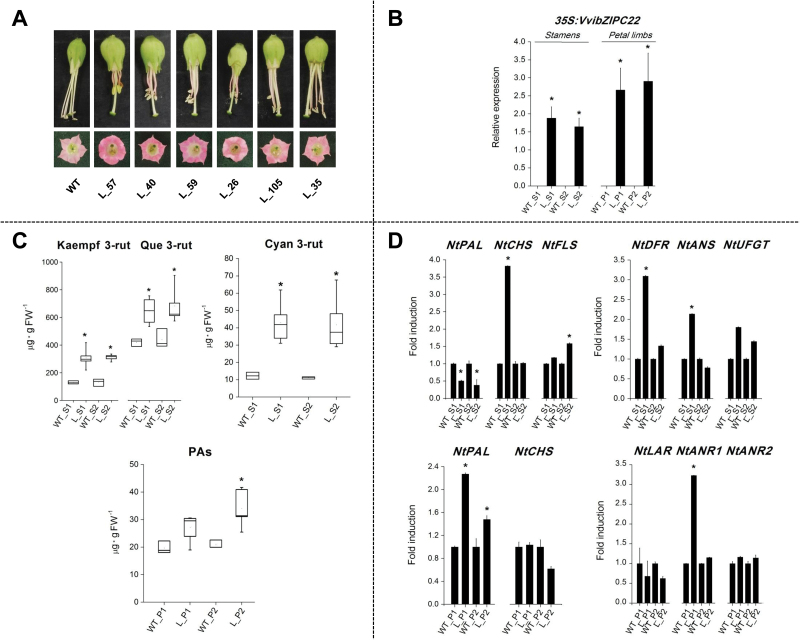
Functional characterization of *VvibZIPC22* in tobacco overexpressing lines. (A) Stamens and corollas of wild-type (WT) plants and transgenic lines (L), and (B) average *VvibZIPC22* relative expression in stamens (S) and petal limbs (P) of three WT and six L at two developmental stages (S1=open flower without pollen, S2= mature flower). (C) Variation of cyanidin 3-rutinoside (Cyan 3-rut), quercetin 3-rutinoside (Que 3-rut), and kaempferol 3-rutinoside (Kaempf 3-rut) in stamens and of proanthocyanidins (PAs) in petal limbs of L and WT plants at the two stages. Anthocyanin and flavonol contents were determined by HPLC-DAD, while PA content was determined by DMACA. (D) Fold induction in gene expression of tobacco flavonoid structural genes between L and WT stamens and petal limbs. Transcript levels in both (B) and (D) were determined by qRT-PCR using gene-specific primers (Supplementary Table S1), calibration against the average expression value in all the lines at each stage, and normalization against *NteIF4a* and *NtUbiquitin* relative expression. Average values with error bars indicating the SE are shown. Asterisks indicate significant changes (*P*<0.05) in the comparison between L and WT plants, tested by a Student’s *t*-test performed on log2-transformed data. PAL, phenylalanine ammonia lyase; CHS, chalcone synthase; FLS, flavonol synthase; LAR, leucoanthocyanidin reductase; ANR, anthocyanidin reductase; DFR, dihydroflavonol 4-reductase; ANS, anthocyanidin synthase; UFGT, UDP-Glc:flavonoid-3-*O*-glucosyltransferase.

Overexpression of *VvibZIPC22* caused line-specific abnormalities, both in the vegetative part (reduced height of the plant, internodes, and size of the leaves) and in the reproductive system (corollas showed visibly increased colour and rounded lobes, and stamens appeared reddish and reduced in size) (Fig. 5A).

As expected, the six lines displayed overexpression of *VvibZIPC22* and with a rather limited variability (±25%) (Fig. 5B). As far as secondary metabolites are concerned (Fig. 5C), striking differences, compared with the WT plants, were obtained looking at the flavonol and anthocyanin content in the stamens at both stages and at the PA content in the petal limbs at the second stage. Interestingly, the increase in flavonoids was paralleled by a significant increase in the expression of several structural genes (Fig. 5D). An induction of *NtCHS*, *NtDFR*, *NtANS*, and *NtUFGT* was observed in the stamens at the first stage together with a significant increase in the content of cyanidin 3-rutinoside (up to 4-fold more than WT plants in both stages), and of *NtFLS* together with an increase of kaempferol 3-rutinoside and quercetin 3-rutinoside in the stamens at the second stage (up to 2.6- and 2-fold more than in WT plants, respectively). In some lines, these results are in agreement with the visibly red colour of the stamens (Fig. 5A). Compared with WT petal limbs, the lines also displayed a higher expression of *NtPAL* and *NtANR1* together with a higher content of PAs at the second stage.

### *In silico* approaches provide new preliminary insights into the *VvibZIPC22* regulatory network

A specific heterodimerization network of bZIP proteins from clade S (clade C in *V. vinifera*) and clade C (clade B in *V. vinifera*) was previously described in Arabidopsis (Ehlert *et al.*, 2006), which is conserved in tobacco (Strathmann *et al.*, 2001) and parsley (Rugner *et al.*, 2001). We thus looked for potential interacting partners of VvibZIPC22 belonging to clade B, by a combination of phylogenetic and co-expression analyses. Our phylogenetic reconstruction (Supplementary Fig. S2) of *VvibZIP* genes from clade B and closely related sequences from other crop species indicated that *VvibZIPB38* and *VvibZIPB09* are more related to members of Rosaceae and Solanaceae, respectively, as is the case for *VvibZIPC22* which shares group C1 with members from the same species.

In *silico* co-expression analyses against a grapevine gene expression compendium using *VvibZIPC22* (VIT_07s0005g01450) as the query gene identified a set of 210 positively co-expressed genes (correlation cut-off=0.68) in 140 contrasts, conditions where *VvibZIPC22* showed the highest modulation (Supplementary Table S3). Of the 210 co-expressed genes, 18 coded for transcription factors including the two bZIP factors, VvibZIPB38 (VIT_14s0030g02200, *r*=0.68) and VvibZIPC14 (VIT_05s0077g01140, *r*=0.69), belonging to clade B and clade C (group C1), respectively. Taken together, the two approaches suggest VvibZIPB38 as a putative heterodimerization partner of VvibZIPC22, and VvibZIPC14 and VvibZIPC22 as mutual interacting partners with VvibZIP38.

**Fig. 6.**
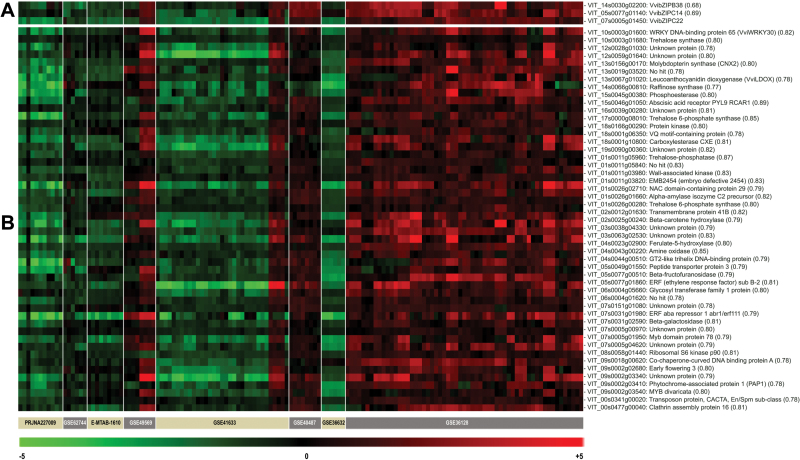
Expression heatmaps of the genes positively correlated with *VvibZIPC22* in 140 contrasts of the grapevine gene expression compendium VESPUCCI (Moretto *et al*., 2016*b*, http://vespucci.colombos.fmach.it/). (A) Expression heatmap of *VvibZIPC22* and of *VvibZIPC14* and *VvibZIPB38*, the only two *bZIP* genes co-expressed with *VvibZIPC22* (Supplementary Table S3). (B) Expression heatmap of the 50 genes showing the highest correlation with the *VvibZIPC22* profile. The uncentred Pearson correlation value between the expression profile of each gene and the *VvibZIPC22* expression profile is shown in brackets. The white vertical lines delimit the different experiments to which the selected contrasts belong. Their accession numbers retrieved from GEO (http://www.ncbi.nlm.nih.gov/geo/) and Arrayexpress (https://www.ebi.ac.uk/arrayexpress/) are indicated on the bar. The colour scale indicates the log2 expression ratio of each test versus the reference condition of the experiment.

In addition, the co-expression analysis provided further preliminary insights into the *VvibZIPC22* gene regulatory network. The list of co-expressed genes was enriched in genes belonging to carbohydrate and amino acid metabolism, to ABA and ethylene signalling, and flower development. In particular, it is worth noting that among the genes with the highest correlation to the *VvibZIPC22* profile, eight code for starch and sucrose metabolism enzymes (one coding for a trehalose phosphatase, one for a trehalose synthase, two for trehalose 6-phosphate synthases, one for an α-amylase precursor, one for a β-galactosidase, one for a glycosyl transferase, and one for a β-fructofuranosidase, *r* from 0.79–0.87), one for a ferulate-5-hydroxylase (*r*=0.80), and one for a leucoanthocyanidin dioxygenase (*r*=0.77), involved in the upper part of the phenylpropanoid pathway and in flavonoid biosynthesis, respectively, and one for an amine oxidase (*r*=0.85), an oxidoreductase that can also participate in phenylalanine metabolism (Fig. 6; Supplementary Table S3).

## Discussion

bZIP factors belongs to a large and diverse gene family identified in several plant genomes. Recently, the bZIP family has also been characterized in grapevine in terms of identification of members and phylogenesis (Liu *et al.*, 2014). Plant bZIPs are involved in the regulation of many different plant processes, including the transcriptional control of amino acid (Hanson *et al.*, 2008; Dietrich *et al.*, 2011) and phenylpropanoid biosynthesis (Heinekamp *et al.*, 2002; Hartmann *et al.*, 2005).

During development, grape flowers and berries accumulate different flavonoids at the organ- and stage-specific level as a consequence of the timely expression of genes necessary for their synthesis. Although flavonoid biosynthesis has been investigated in depth also in grapevine, its regulation is not completely elucidated (Hichri *et al.*, 2011a).

R2R3-MYB factors are known to be the key regulators of this transcriptional network; however, there are examples supporting the involvement of bZIP proteins, acting synergistically with MYB and other factors, in the control of light-induced flavonoid biosynthesis (Hartmann *et al.*, 2005). The most studied is ELONGATED HYPOCOTYL5 (HY5), a bZIP associated with *AtCHS* and *PFG1/MYB12* activation and accumulation of ﬂavonoids upon light treatment (Stracke *et al.*, 2010).

Interestingly, we have recently identified a gene encoding a bZIP factor, namely *VvbZIP22*, located on chromosome 7 within a QTL region specific for kaemperol content in mature berries (Malacarne *et al.*, 2015), and therefore a good candidate as a regulator of the flavonol pathway. Starting from this evidence, in this study we further characterized the involvement of VvbZIP22, here renamed VvibZIP22C, in the fine regulation of flavonol biosynthesis.

By phylogenetic reconstruction, we showed that *VvibZIPC22*, together with *VvibZIPC14* and *VvibZIPC37*, forms a group of the tree and is the potential orthologue of previously characterized bZIPs from flowering species such as *N. tabacum* (*NtBZI-3* and *NtBZI-4*)*, P. crispum* (*PcCPRF6*), *A. thaliana* (*AtbZIP53*), and *S. lycopersicum* (*SlbZIP04*, also known as *LebZIP2*). These genes are associated with flower development (Strathmann *et al.*, 2001), are induced by salt and light treatments (Seong *et al.*, 2008; Li *et al.*, 2015), mediate the sugar-dependent level of specific amino acids during starvation (Dietrich *et al.*, 2011), and are involved in the regulation of phenylpropanoid pathway genes by binding to specific *cis*-regulatory elements (Rugner *et al.*, 2001; Heinekamp *et al.*, 2002).

*VvibZIPC22* expression was particularly high in the flower, the same organ in which kaempferol and quercetin accumulate the most, and its profile during Pinot Noir berry development resembled that of *VviMYBF1* (Czemmel *et al.*, 2009) and *VviFLS1* (Downey *et al.*, 2003), encoding the key regulators of flavonol biosynthesis in grapes. This result was a further indication that VvibZIPC22 is involved in the fine regulation of flavonol biosynthesis.

Flavonols, among flavonoids, are known to be modulated by UV light and function as UV protectants (Teixeira *et al.*, 2013). The light-induced flavonol biosynthesis is guided by the expression of *VviMYBF1* and *VviFLS1*, genes containing light regulatory *cis*-acting elements in their promoters, recognized by MYB and bZIP factors (Czemmel *et al.*, 2009). Based on this knowledge, we examined whether VvibZIPC22 is also involved in UV light-induced flavonol biosynthesis. Indeed, *VvibZIPC22* appeared to be a light-inducible gene, being up-regulated within 10h of UV light treatment of Chardonnay leaves, together with *VviMYBF1* and *VviFLS1.* A similar result was also previously observed for *VviMYBF1* and *VviFLS1* in Chardonnay cell cultures (Czemmel *et al.*, 2009). The induction of these genes was accompanied by flavonol accumulation starting at 24h and peaking at 72h after treatment. The analysis of the promoter region of *VvibZIPC22* indicated that its light-induced expression might be due to the presence of MRS and ACS light regulatory *cis*-acting elements, the same as found in the promoter sequence of *VviMYBF1* and *VviFLS1* (Czemmel *et al.*, 2009) and conferring light responsiveness to *AtCHS* and *AtFLS* (Hartmann *et al.*, 2005). Whether *VvibZIPC22* light induction is mediated by the grapevine HY5 orthologue and by MYB factors, however, remains to be elucidated.

Overexpression of *VvibZIPC22* in tobacco and transient promoter assays in Chardonnay cell cultures were then adopted as complementary approaches to investigate the role VvibZIPC22 further *in vivo*, demonstrate a causal link between VvibZIPC22 and flavonol biosynthesis, and gain insights into its putative regulatory network.

By these experiments, we provided evidence that VvibZIPC22 impacts the entire flavonoid pathway. Indeed, the tobacco overexpressing lines displayed a significantly higher content of the flavonols kaempferol and quercetin 3-rutinoside, the anthocyanin cyanidin 3-rutinoside, and the PAs in flowers, at the organ- and stage-specific level. However, due to the type of assay, we cannot completely exclude that the monitored increase in PAs corresponded to a change of the mean degree of polymerization (mDP), rather than a net increase in the absolute amount of PAs. These biochemical changes were accompanied by a significant induction of several flavonoid structural genes. Noteworthy is the ectopic expression of *VvibZIPC22* that caused in most lines a visibly stronger red colouring of the stamens and corolla but not of the vegetative organs, as observed in tobacco flowers overexpressing *VviMYB5a* and *VviMYB5b* (Deluc *et al.*, 2006, 2008). Conversely, the ectopic expression of *VlMYBA1-2* triggered *de novo* production and storage of anthocyanins in all vegetative organs of the grapevine transgenic lines, attesting to a major effect of the gene on anthocyanin biosynthesis (Cutanda-Perez *et al.*, 2009). As proposed in the model derived from the functional characterization of *VviMYB5b* (Hichri *et al.*, 2011b), we can speculate that similarly VvibZIPC22 interacts with endogenous partners in the flower, where the anthocyanin pathway is already active (corolla), or induces *de novo* anthocyanin biosynthesis by interacting with other endogenous partners of the organs where the pathway is not yet active (stamens).

The transient promoter assays confirmed that VvibZIPC22 acts on different points of the flavonoid biosynthetic pathway, working either alone or in combination with other factors. VvibZIPC22 was capable of directly activating the flavonol-specific gene *VviFLS1* and the flavan-3-ol-specific gene *VviANR*, as well as the genes of the general flavonoid pathway, *VviCHS3* and *VviCHI*, by putatively binding to an ACGT-containing element identified in a conserved position in their promoters. Interestingly, VvibZIPC22 also increased the transactivation potential of known regulators of the general and specific flavonoid pathway genes, with the exception of the VviMYBA2–MYC1 complex which is specific for the regulation of the anthocyanin gene *VviUFGT* (Hichri *et al.*, 2010). This result, contrasting with findings in tobacco, might be explained by a limit in the transactivation capacity of the *VviUFGT* promoter in this assay. Here, the availability of specific regulators is much higher than in reality and therefore there is no possibility of further activation by other factors such as VvibZIPC22.

Heterodimerization is a typical mechanism for regulating bZIP factor activity, and the formation of specific bZIP pairs is essential to exert their function (Llorca *et al.*, 2014). In particular, in Arabidopsis (Ehlert *et al.*, 2006), bZIPs from clade S (clade C in *V. vinifera*, to which VvibZIPC22 belongs) form specific heterodimers with bZIPs from clade C (clade B in *V. vinifera*). This heterodimeric complex is also conserved in tobacco (Strathmann *et al.*, 2001) and parsley (Rugner *et al.*, 2001). Accordingly, the putative heterodimerization partners of VvibZIPC22 were searched for within the bZIPs of clade B (Liu *et al.*, 2014). From phylogenetic and co-expression results, we obtained an indication of VvibZIPB38 as a putative heterodimerization partner of VvibZIPC22, to be experimentally validated.

The co-expression analysis also gave further insights into the *VvibZIPC22* putative regulatory network. Remarkably, 40% of the 210 genes strongly co-expressed with *VvibZIPC22* fell within the QTL regions recently associated with the fine regulation of anthocyanin and flavonol content in mature berries (Costantini *et al.*, 2015; Malacarne *et al.*, 2015). This percentage represents a significant enrichment over a random distribution of these genes along the genome, supporting their involvement in flavonoid metabolism. In addition, we noted that several genes with the highest correlation to the *VvibZIPC22* profile coded for enzymes related to glucose metabolism. Interestingly, in Arabidopsis the expression of *bZIP* genes from class S1 was shown to be regulated by sugars and therefore to participate in a sugar-dependent control of target genes (Hanson *et al.*, 2008).

**Fig. 7.**
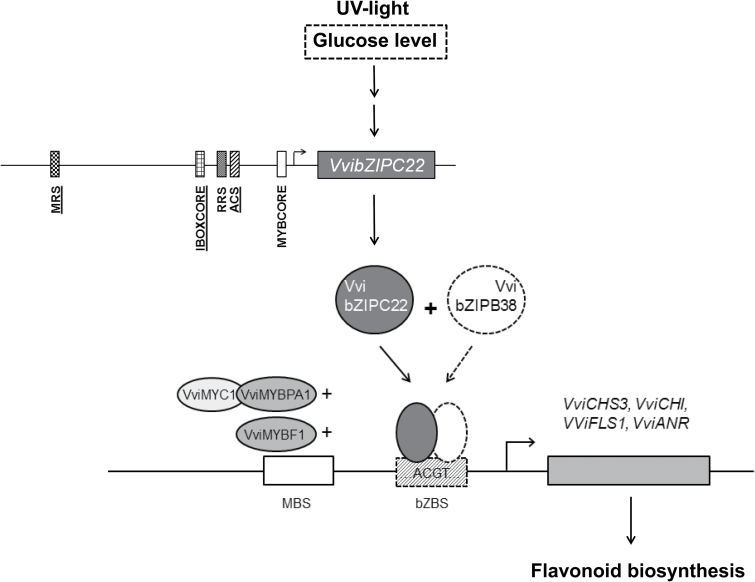
Hypothetical working model of VvibZIPC22 as a regulator of the grapevine flavonoid pathway. UV light and/or the glucose level activate the transcription of *VvibZIPC22* whose promoter contains light response *cis*-acting elements (underlined). Upon heterodimerization with VvibZIPB38 and, in specific cases, by interaction with known MYB and bHLH factors, VvibZIPC22 regulates the expression of several flavonoid pathway genes, including *VviCHS3*, *VviCHI*, *VviFLS1*, and *VviANR*, by recognizing an ACGT-containing element in their promoters. The involvement of the elements in dashed boxes needs experimental validation. For the description of the *cis*-acting elements in the *VvibZIPC22* promoter, refer to the legend of Fig. 4. MBS, MYB-binding site; bZBS, bZIP-binding site; CHS3, chalcone synthase 3; CHI, chalcone isomerase; FLS, flavonol synthase; ANR, anthocyanidin reductase.

Overall the results of our study point to a role for VvibZIPC22 in the regulation of different branches of the flavonoid pathway. In our hypothetical model, UV light and perhaps the glucose level promote the transcription of *VvibZIPC22*, which then regulates the expression of several genes of the flavonoid pathway, either alone or in combination with other factors (Fig. 7).

### Conclusions

We have functionally characterized VvibZIPC22, a grapevine bZIP factor belonging to clade C in the phylogenetic reconstruction of the grapevine *bZIP* gene family. Our results indicated that, upon UV light stimulus, VvibZIPC22 regulates the content of different flavonoids, acting on promoters of general and specific genes of the pathway, alone or in combination with other factors. Altogether, our data provide new insights into the transcriptional control of the grapevine flavonoid pathway.

## Supplementary data

Supplementary data are available at *JXB* online.

Table S1. Primers used in qRT-PCR analyses of grapevine and tobacco samples.

Table S2. Putative ACGT-containing elements in the promoters of the grapevine flavonoid pathway genes tested in transient reporter assays.

Table S3. Results of co-expression analysis obtained with *VvibZIPC22* as input query.

Figure S1. Profiles of *VvibZIPC22* relative expression and of anthocyanin 3-monoglucosides and flavan-3-ol monomers during Pinot Noir berry development.

Figure S2. Phylogenetic tree of clade B VvibZIP factors and of their putative orthologues.

Dataset S1. Codon-based alignments of CDS sequences of clade B *VvibZIP* genes and of their putative orthologues.

Dataset S2. Codon-based alignments of CDS sequences of clade C *VvibZIP* genes and of their putative orthologues.

Supplementary Data
