# Supplementary material for: The grapevine VvibZIPC22 transcription factor is involved in the regulation of flavonoid biosynthesis
Source: J Exp Bot. 2016 May 18;67(11):3509–22. doi: 10.1093/jxb/erw181 (PMC4892739; doi:10.1093/jxb/erw181)
Supplement: Supplementary Data [file supp_67_11_3509__index.html]

The grapevine VvibZIPC22 transcription factor is involved in the regulation of flavonoid biosynthesis — The grapevine VvibZIPC22 transcription factor is involved in the regulation of flavonoid biosynthesis — Supplementary Data 

# The grapevine VvibZIPC22 transcription factor is involved in the regulation of flavonoid biosynthesis

## Supplementary Data

Data files

- supplementary\_table\_S2\_Figures\_S1\_S2.docx - Supplementary Data
- supplementary\_table\_S1\_and\_S3.xlsx - Supplementary Data
- supplementary\_dataset\_S1.fasta - Supplementary Data
- supplementary\_dataset\_S2.fasta - Supplementary Data
