# Supplementary material for: The grapevine VvibZIPC22 transcription factor is involved in the regulation of flavonoid biosynthesis
Source: J Exp Bot. 2016 May 18;67(11):3509–22. doi: 10.1093/jxb/erw181 (PMC4892739; doi:10.1093/jxb/erw181)

**The grapevine VvibZIPC22 transcription factor is involved in the regulation of flavonoid biosynthesis**

Giulia Malacarne, Emanuela Coller, Stefan Czemmel, Urska Vrhovsek, Kristof Engelen, Vadim Goremykin, Jochen Bogs, and Claudio Moser

**Table S2.** Putative ACGT-containing elements in the promoters of the grapevine flavonoid pathway genes tested in transient reporter assays. Elements were identified by scanning the first 1000 base pairs (bp) from the transcriptional start site (+1) of the promoter region and their position is indicated in brackets. The conserved ACGT core is highlighted in bold. Abbreviations: CHS= Chalcone Synthase; CHI= Chalcone Isomerase; FLS= Flavonol Synthase; ANR= Anthocyanidin Reductase; UFGT= UDP-Glc:Flavonoid-3-O-glucosyltransferase; nd=not detected.

| **Gene abbreviation** | **Accession number^*^** | **Promoter region from the transcriptional start site (+1)** | | | | | | | |
| --- | --- | --- | --- | --- | --- | --- | --- | --- | --- |
|  |  | 0…-50 bp | -51…-100 bp | -101…-150 bp | >-150 bp | | | | |
| *VviCHS1* | AB015872 | nd | nd | nd | CAT**ACGT**CAC (-678) | | GTC**ACGT**ACA (-775) |  |  |
| *VviCHS2* | AB066275 | nd | nd | nd | nd | | |  |  |
| *VviCHS3* | AB066274 | nd | AGC**ACGT**GAC  (-96) | GTC**ACGT**GCC (-142) | nd | | |  |  |
| *VviCHI* | X75963 | ATA**ACGT**CGC (-35) | nd | ACA**ACGT**GCC (-127) | CGT**ACGT**GCA  (-380) | | TGT**ACGT**ACA (-722) |  |  |
| *VviFLS1* | FJ948478 | nd | nd | CAC**ACGT**CAC (-145) | nd | | | |  |
| *VviANR* | CAD91911 | nd | nd | CTC**ACGT**GCT (-114) | ATA**ACGT**CGG (-458) | ACC**ACGT**GGT (-643) | | |  |
| *VviUFGT* | AY955269 | nd | nd | nd | nd | | | |  |

**^*^** Accession number of the National Center for Biotechnology Information (NCBI) database ([www.ncbi.nlm.nih.gov](http://www.ncbi.nlm.nih.gov)).


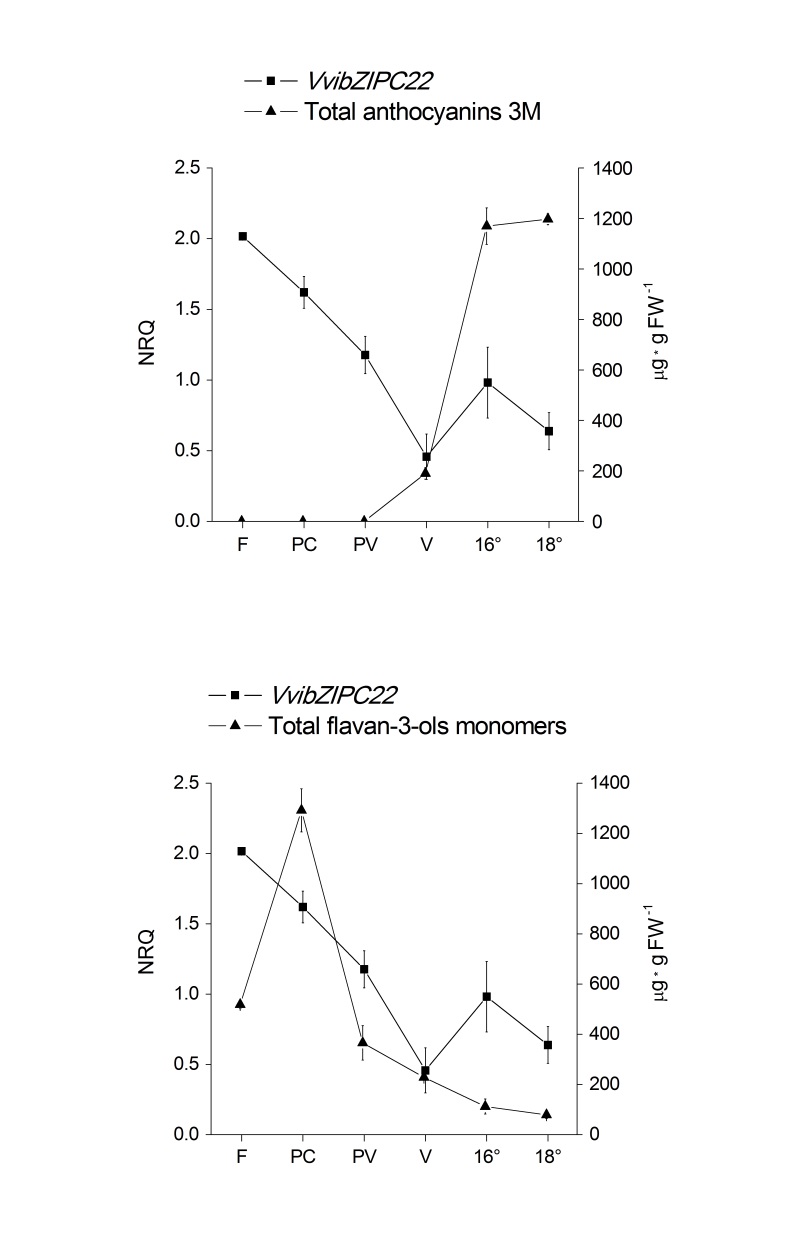
**Figure S1.** Profiles of *VvibZIPC22* relative expression and of anthocyanin 3-monoglucosides and flavan-3-ols monomers during Pinot Noir berry development. Transcript levels (NRQs) were determined by qRT-PCR using gene-specific primers (Table S1), calibration against the average expression value in all the stages and normalization against the *VviGADPH* and *VviACTIN* relative expression. Total anthocyanins 3M and total flavan-3-ols monomers correspond to the sum of the content of cyanidin, peonidin, delphinidin, petunidin, and malvidin 3M in the first case, and of catechin, epicatechin, and gallocatechin in the other, as were the one detected by HPLC-DAD analysis in the flower extract (flowering stage), the total berry extracts (pepper corn and pre-véraison stages) and the skin extracts (from véraison to maturity stages). Each value corresponds to the mean and standard error of three different biological replicates. F, flowering (50% opened flowers, E-L 23), P, pepper corn (E-L 29), PV, pre-véraison (hard green berries, E-L 33), V, véraison (50% coloured berries, E-L 35), 16°, post-véraison (berries at 16° Brix, E-L 36); 18°, maturity (berries at 18° Brix, E-L 38); NRQ= Normalized Relative Quantity; 3M= 3-monoglucosides; FW= fresh weight.

**Figure S2.** Phylogenetic tree of clade B *VvibZIP* factors and of their putative orthologs. A Bayesian analysis was performed employing the MPI version of Phylobayes (version 1.4) and a general specification of CAT+GTR+G4 model. Two chains were run on a 1335 positions-long curated codon-based alignment under each model for each of the data sets. The tree was built for each dataset based on the two chains, discarding the first 2500 cycles as burning, which was sufficient for ML parameter values to maximize in all analyses. Parameter values were then sampled every cycle thereafter till the 5000th cycle. Bootstrap values are shown next to the nodes. Gene names with their accession numbers are the following: *AmTriCPRF2-like* (XM_011623312), *AtbZIP09* (NM_122389), *AtbZIP10* (NM_178959), *AtbZIP25* (NM_001203162), *AtbZIP63* (NM_122760), *CcCPRF2-like* (XM_006432867), *CmCPRF2-like* (XM_008457728), *CsCPRF2-like* (XM_004139185), *EgCPRF2-like* (XM_010937737), *G/HBF-1* (NM_001249370), *MdCPRF2-like* (XM_008347543), *NtBZI-1* (AY061648), *PaCV000596.1_f3* (CV000596), *PcCPRF2* (X58577), *PtCPRF2-like* (XM_002319036), *PxbCPRF2-like* (XM_009366320), *SiCPRF2-like* (XM_011093646), *SlbZIP06* (Solyc01g097330), *StCPRF2-like* (XM_006347686), *TcCPRF2-like* (XM_007030512), *VvibZIPB09* (VIT_04s0008g02750), *VvibZIPB21* (VIT_07s0141g00170), *VvibZIPB38* (VIT_14s0030g02200).


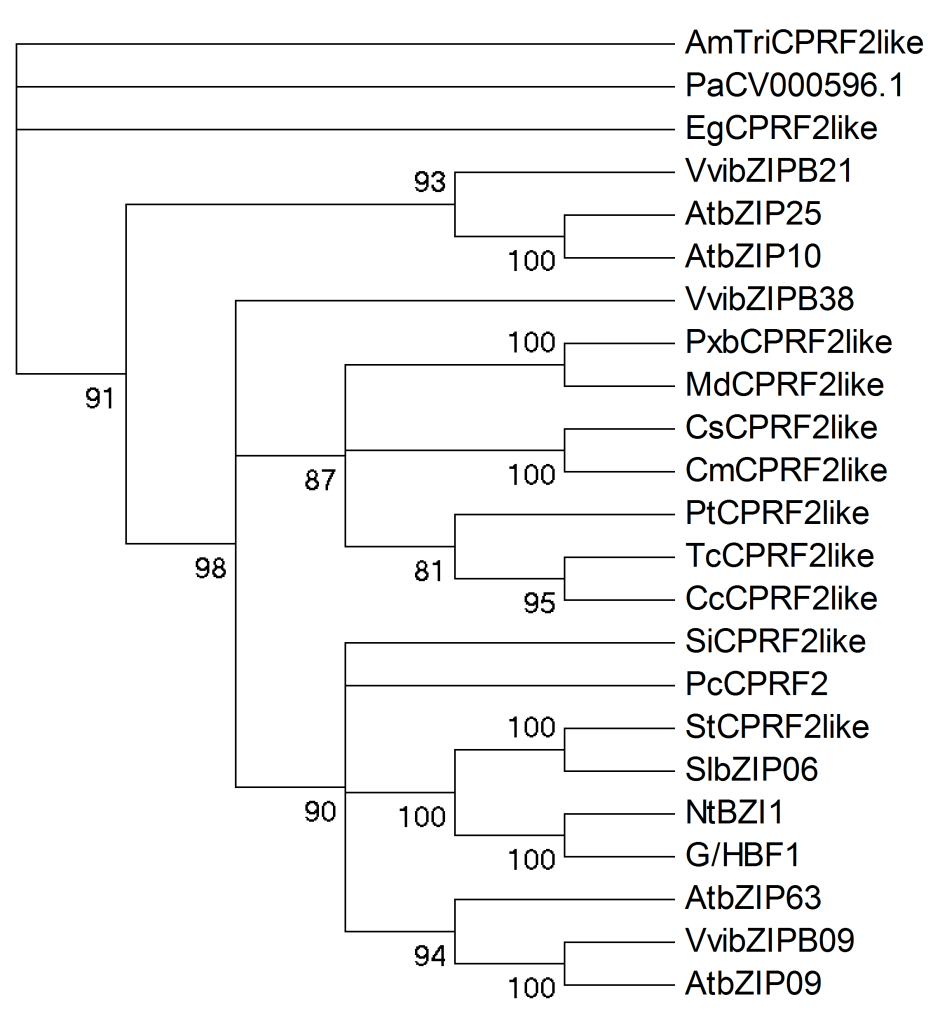

Supplement: Supplementary Data [file supp_erw181_supplementary_table_S2_Figures_S1_S2.docx]
